# PPGTempStitch: A MATLAB Toolbox for Augmenting Annotated Photoplethsmogram Signals

**DOI:** 10.3390/s21124007

**Published:** 2021-06-10

**Authors:** Qunfeng Tang, Zhencheng Chen, Carlo Menon, Rabab Ward, Mohamed Elgendi

**Affiliations:** 1 School of Electronic Engineering and Automation, Guilin University of Electronic Technology, Guilin 541004, China; tangqunfeng@mails.guet.edu.cn (Q.T.); chenzhcheng@guet.edu.cn (Z.C.); 2 Department of Electrical and Computer Engineering, University of British Columbia, Vancouver, BC V6T 1Z4, Canada; rababw@ece.ubc.ca; 3 Biomedical and Mobile Health Technology Laboratory, Department of Health Sciences and Technology, ETH Zurich, 8008 Zurich, Switzerland; carlo.menon@hest.ethz.ch; 4 School of Mechatronic Systems Engineering, Simon Fraser University, Surrey, BC V3T 0A3, Canada

**Keywords:** PPG synthesis, pleth augmentation, PPG generators, imbalanced PPG, PPG augmentation, enlarging time-series health data, PPG in low-resource clinical settings, PPG lengthening, PPG signal extension, upsizing existing PPG databases

## Abstract

An annotated photoplethysmogram (PPG) is required when evaluating PPG algorithms that have been developed to detect the onset and systolic peaks of PPG waveforms. However, few publicly accessible PPG datasets exist in which the onset and systolic peaks of the waveforms are annotated. Therefore, this study developed a MATLAB toolbox that stitches predetermined annotated PPGs in a random manner to generate a long, annotated PPG signal. With this toolbox, any combination of four annotated PPG templates that represent regular, irregular, fast rhythm, and noisy PPG waveforms can be stitched together to generate a long, annotated PPG. Furthermore, this toolbox can simulate real-life PPG signals by introducing different noise levels and PPG waveforms. The toolbox can implement two stitching methods: one based on the systolic peak and the other on the onset. Additionally, cubic spline interpolation is used to smooth the waveform around the stitching point, and a skewness index is used as a signal quality index to select the final signal output based on the stitching method used. The developed toolbox is free and open-source software, and a graphical user interface is provided. The method of synthesizing by stitching introduced in this paper is a data augmentation strategy that can help researchers significantly increase the size and diversity of annotated PPG signals available for training and testing different feature extraction algorithms.

## 1. Introduction

Photoplethysmography is a technology that optically detects changes in the blood volume of microvascular tissue beds. This technology, which can obtain a wealth of information about the cardiovascular system, has received extensive attention from scientists with different backgrounds, as it is non-invasive and can be continuously monitored. It is also used in devices like smartphones and wearable devices for health monitoring and primary health checks [1,2].

Photoplethysmography can be used to evaluate heart rate [3,4], blood oxygen saturation [5], respiration rate [6], hypertension [7,8], the ankle–brachial pressure index [9], vascular aging [10,11], and other cardiovascular parameters.

When analyzing photoplethysmogram (PPG) signals, researchers usually need to extract the features in the time domain, such as the locations and amplitudes of the PPG systolic peaks and the onsets [12]. Therefore, the accuracy of the feature extraction algorithm will affect the accuracy of the analysis results. To evaluate the performance of PPG time domain feature extraction algorithms, a large number of PPG signals with different sampling frequencies, noise levels, morphologies, and heart rhythms are required. However, few publicly accessible annotated PPG signals exist. The MIMIC database [13] contains 64 annotated PPG signal records, the length of which is one hour, and only the onsets of the PPG waveforms are annotated. Additionally, the IEEE TBME Respiratory Rate Benchmark dataset has 42 annotated PPG signal records, each eight minutes long [14], but only the systolic peaks are annotated in these records. Therefore, this study aimed to develop a toolbox to generate PPGs due to the high interest in research related to PPG signal analysis and app development.

Some mathematical models have been used to generate PPGs. In [15], a nonlinear model was used to generate reference PPG to clean PPGs before extracting features. Another method used the two Gaussian functions to model a single pulse to generate PPGs [16]; this method used the average of two autoregressive moving average models to approximate the parameters of the pulse model. Similarly, another method using a single pulse modeled by a log-normal and two Gaussian functions was applied in [17]. The beat-to-beat intervals were extracted from the RR interval in the electrocardiogram signal, and each PPG pulse was then connected according to the RR interval. Furthermore, the authors’ previous work utilized a Two Gaussian Functions model based on circular motions to generate regular and irregular PPGs [18,19]. However, these methods [15,16,17,18,19] were focused on the morphology of the PPG, and no annotations were provided.

In this study, a MATLAB toolbox called “PPGTempStitch” was developed to stitch short-time annotated PPG templates together to generate a new long-time PPG signal for any sampling frequency. Stitching technology has been widely used in image processing [20,21,22]. To our knowledge, this present study is the second attempt to apply the stitching idea to time-series PPG signals. The first attempt to stitch time-series PPG signals was discussed in [23]. The “PPGTempStitch” toolbox generated by this study is an open-source package designed to use existing routines in MATLAB 2020a for reproducibility.

## 2. Materials and Methods

**Figure 1:**
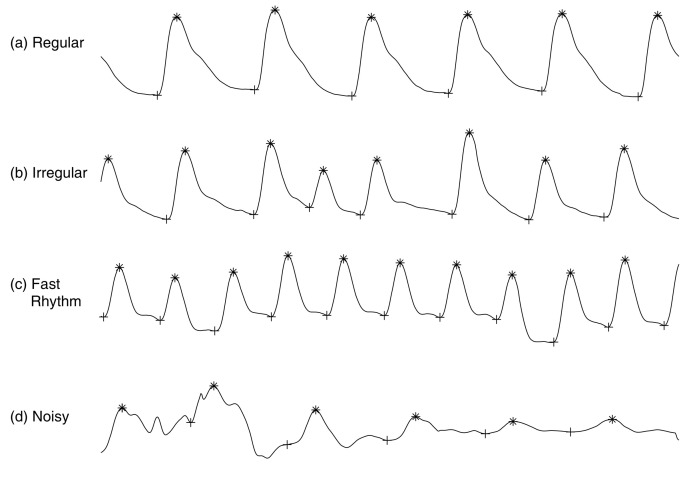
Four types of PPG templates. The regular, irregular, fast-rhythm, and noisy templates are (**a**–**d**), respectively. The “*” and “+” in (**a**–**d**) refer to the annotated systolic peak and onset, respectively.

**Figure 2:**
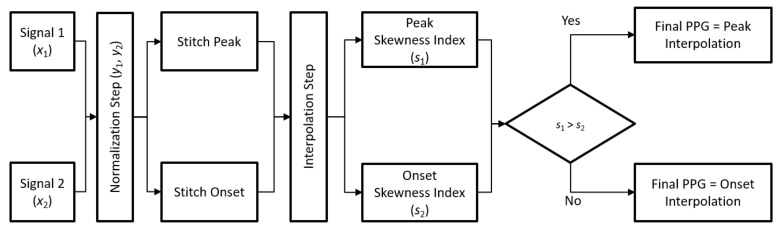
Flowchart for stitching two PPGs together. The “Peak” refers to the systolic peak.

To evaluate the performance of PPGTempStitch when stitching different PPG types together, four types of normalized PPG templates were used for testing: regular PPG, irregular PPG, fast-rhythm PPG, and noisy PPG. Regular PPG occurs when the PPG rhythm is regular and the mean heart rate is within 59–99 beats per minute. Irregular PPG occurs when the mean heart rate is within 59–99 and the PPG rhythm is irregular. Fast-rhythm PPG occurs when the PPG rhythm is irregular but the mean heart rate is higher than 100. Lastly, noisy PPG occurs when the PPG segments contain noise. Figure 1 shows the dataset that was used in this study, these PPG templates came from different subjects in the MIMIC III database. The templates, which have already been normalized, were recorded at a 125 Hz sampling frequency. Figure 2 shows the flow chart for the steps used to stitch two PPGs ($x_{1}$ and $x_{2}$). These steps were normalization, stitching, interpolation, and method selection.

### 2.1. Normalization

PPGs may have different amplitudes. To enable comparison of different PPGs, they are usually normalized in pre-processing. In the present study, min-max and z-score normalization were both utilized to scale the two PPG segments before they were stitched together; this ensured that the amplitudes of the PPGs were consistent.

Min-max normalization was used to scale PPGs in the 0–1 range, as follows:

(1) y=(x−min(x))/(max(x)−min(x)),

where *x* is an original PPG signal, and *y* is the normalized PPG signal. The other normalization method, called the z-score, was used to center PPGs with a mean of 0 and scaled them to have a standard deviation of 1, as follows:

(2) $$y=\frac{x-\mu }{\sigma },$$

where *x* is an original PPG signal; μ and σ are the mean and standard deviation of *x*, respectively; and *y* is the normalized PPG signal. In the current study’s toolbox, the two normalization methods could not be used at the same time. The normalized PPGs were named “$y_{1}$” and “$y_{2}$”, which correspond to the first PPG ($x_{1}$) and second PPG ($x_{2}$), respectively.

### 2.2. Stitching

A major challenge for stitching two PPG signals together is choosing the stitching point, as the amplitude of the end point of the first PPG ($y_{1}$) is always different from that of the starting point of the second PPG ($y_{2}$). In this case, two signals were stitched together based on the systolic peak or the onset—the main two features of the PPG signal in the time domain. The difference between these two methods of selecting the stitching point was as follows:

- **Based on the systolic peak.** For the first PPG, the stitching point was the last systolic peak, while the stitching point of the second PPG was its first systolic peak.
- **Based on the onset.** For the first PPG, the stitching point was the last onset, while the stitching point of the second PPG was its first onset.

**Figure 3:**
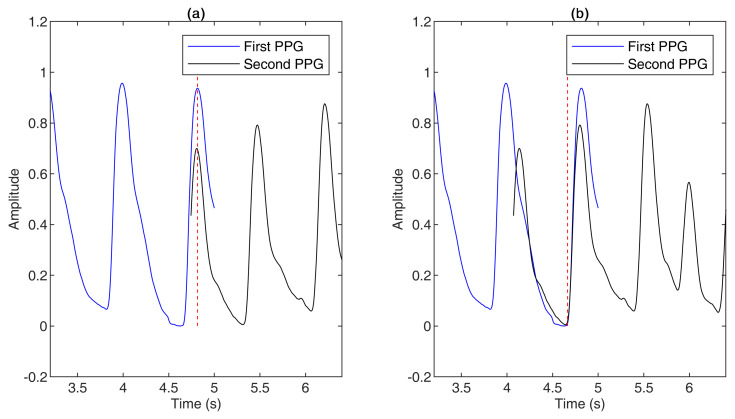
Comparison of stitching two PPG signals together based on the systolic peak or based on the onset. (**a**) First and second PPGs aligned based on the systolic peak. (**b**) First and second PPGs aligned based on the onset. The red dotted lines in (**a**,**b**) are the aligned lines of the first and second PPGs.

The two PPGs were then aligned in time based on the stitching point. The segment after the last onset of the first PPG and the segment before the first onset of the second PPG were discarded. Figure 3 compares stitching based on the systolic peak with stitching based on the onset.

### 2.3. Interpolation

The value of the stitching point in the first PPG ($y_{1}$) may be different from that in the second PPG ($y_{2}$). Therefore, to smooth the stitching point, cubic spline data interpolation was used [24]. Cubic spline interpolation involves a spline in which each piece is a third-degree polynomial specified by its values and first derivatives at the end points of the corresponding domain interval.

The interpolation involved a total of 20 samples: the stitching point, nine samples before the stitching point, and 10 samples after the stitching point. To express these more clearly, the PPG segments involved in the interpolation were named “$y_{t}$” and those that were used after the interpolation were named “*z*”.

In this study, the first five and last five samples of $y_{t}$ were used to fit the interpolation function, and the values of the 10 other samples in the middle of $y_{t}$ were replaced by the values of the samples in *z*.

### 2.4. Stitching Beat Selection

As skewness is the optimal signal quality index (SQI) of PPGs [23], the skewness of the previous, stitched, and next beats were calculated. One PPG beat began with the onset of a beat and ended with the onset of the following beat. For the stitching method based on the systolic peak, the stitching beat was the beat where the stitching point was located. For the stitching method based on the onset, the stitching beat was the beat following the stitching point. The previous beat was the beat before the stitching beat, while the next beat was the beat following the stitching beat. To examine a PPG beat, the skewness was applied as follows:

(3) $$k=\frac{1}{N}\sum_{i=1}^{N}{\left[\left(z\left(i\right)-\mu \right)/\sigma \right]}^{3},$$

where μ and σ are the mean and standard deviation of the beat, respectively; z(i) is the value of the samples; and *N* is the number of samples in the beat. The skewness index was calculated as follows:

(4) $$s=k_{previous}+k_{stitching}+k_{next},$$

where $k_{previous}$, $k_{stitching}$, and $k_{next}$ are the skewness of the previous, stitched, and next beats, respectively. In this study, “$s_{1}$” was the skewness index of the stitching method based on the systolic peak, and “$s_{2}$” was the skewness index of the stitching method based on the onset.

The skewness index was used to evaluate the quality of stitching, and the stitching method with a higher skewness index was considered better than that with a lower skewness index. To improve the signal quality, the result of the stitching method with a higher skewness index was chosen as the final output in this study.

**Figure 4:**
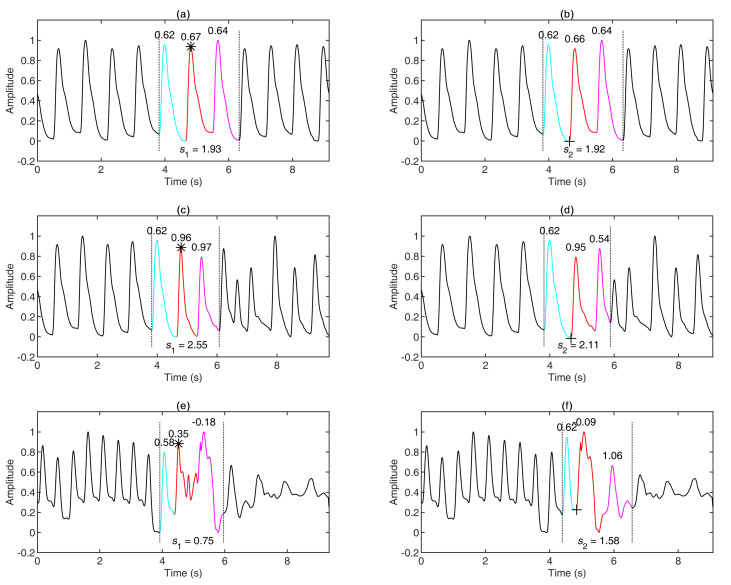
Three examples of stitching results. (**a**,**b**) are the results of stitching two regular PPGs together based on the systolic peak and the onset, respectively. (**c**,**d**) are the results of stitching a regular PPG with an irregular PPG based on the systolic peak and the onset, respectively. (**e**,**f**) are the results of stitching a noisy PPG with an irregular PPG based on the systolic peak and the onset, respectively. The stitching points are denoted as “*” in (**a**,**c**,**e**) and as “+” in (**b**,**d**,**f**). The cyan, red, and magenta parts are the previous, stitching, and next beats, respectively. The numbers above the curve represent the skewness of the beat. The “$s_{1}$” and “$s_{2}$” refer to the skewness index in the stitching methods based on the systolic peak and the onset, respectively.

Table 1 shows the skewness of the aforementioned three beats and the skewness index in different combinations. Figure 4 provides three examples of stitching results. Figure 4a,b are the results of stitching a regular PPG with another regular PPG based on the systolic peak and the onset, respectively; Figure 4c,d are the results of stitching a regular PPG with an irregular PPG based on the systolic peak and the onset, respectively; and Figure 4e,f are the results of stitching a fast-rhythm PPG with a noisy PPG based on the systolic peak and the onset, respectively. The skewness indices in Figure 4a,c,f are greater than those in Figure 4b,d,e, respectively. The differences in the signal quality of the PPG waveforms between Figure 4a,b and between Figure 4c,d are not very obvious, but the signal quality of the PPG waveform in Figure 4f is obviously better than that in Figure 4e. The length of the stitched PPG was less than the sum of the lengths of two PPGs.

PPG templates were augmented to generate new PPG signals (see Figure 4), which can be helpful when the training PPG sample size is small (e.g., 10 PPG recordings of subjects with a specific disease). The introduced strategy stitched templates in a random fashion, similar to combining different images using the data augmentation strategies that are utilized by 2D image processing to generate new images [25]. The math behind data augmentation transformations in 2D signal processing is usually simple (e.g., scaling, translating). The present study opened this area of augmentation for 1D signal processing using PPG signals, and the results were impressive. To the authors’ knowledge, this was the first study of 1D signal analysis to discuss stitching as an augmentation step to generate annotated time-series health data, such as PPGs.

### 2.5. Annotations

Annotations are the labeled PPG waveform, such as a systolic peak, a diastolic peak, an onset, and a dicrotic notch in the time domain [26,27]. For the toolbox developed in this study, the onset and systolic peak were supported. Additionally, the annotations could be merged after the PPGs were stitched together. For the first PPG, the annotations before the stitching point were reserved, while those after the stitching point were discarded. For the second PPG, the annotations before the stitching point were discarded, while those after the stitching point were merged with the reserved annotations in the first PPG according to their positions relative to the stitching point.

Since the interpolation step changed the values of the stitching point, the previous four samples, and the following five samples, the annotation at the stitching point needed to be corrected. For the stitching method based on the systolic peak, the systolic peak at the stitching point was corrected to the maximum value of the 10 samples after interpolation. For the stitching method based on the onset, the onset at the stitching point was corrected to the minimum value of the 10 samples after the interpolation.

### 2.6. Onset and Systolic Peak Detection Algorithms

To obtain the optimal onset and systolic peak extraction algorithm, three pulse wave extraction algorithms were compared, as follows:

- Method I [28] used signal derivative, Hilbert transform, amplitude thresholding, and slope-reversal based approaches. The steps were as follows:
  – PPGs were filtered using a sixth-order Butterworth low-pass filter with a cutoff frequency of 15 Hz.
  – The Hilbert transform was applied to the second derivative of the PPG signal.
  – In the Hilbert transform data, the region greater than 50% amplitude was selected. The slope reversal points within these areas were determined as the maximum peaks.
  – According to the maximum peak position, the Hilbert transform PPG data’s left and right neighborhood samples were tested to determine the primary and next zero-crossing point.
  – Based on each zero-crossing point, 30 samples of the actual PPG data were used to create a search zone.
  – **Onset detection:** According to the left zero-crossing point, samples with slope reversal characteristics were identified as the pulse’s onset.
  – **Systolic peak detection:** At the right zero-crossing point, only those samples with the slope reversal characteristics were identified as the systolic peak.
- Method II [29] first extracted the initial peaks, and classified them as true and false peaks. For each false peak, an algorithm was used to relocate the true peak. The steps were as follows:
  – **Systolic peak detection:**
    ∗ The missing data points or data points with values greater than 20 times the PPG’s median waveform height were defined as outliers. The outlier data points that lasted for less than 0.2 s were linearly interpolated, and the result was denoted as w0.
    ∗ w0 was linearly detrended, and its power spectrum density was computed using a fast Fourier transform. The maximum power spectrum in the 0.8–3.0 Hz range was the heartbeats’ frequency, while the average beat-to-beat interval was its reciprocal value.
    ∗ The waveform w0 was smoothed using a center median filter with a window size set to one-fifth of the estimated beat-to-beat interval, followed by a center moving-average filter with the same window size to generate w1.
    ∗ w2 was generated by the smoothed w1 passed through a third-order, low-pass Butterworth filter with a cutoff frequency of one-and-a-half times the estimated heartbeat frequency.
    ∗ The baseline *b* of the PPG waveform was generated by applying a center moving-average filter to waveform w2, with a window size set to 1.5 of the estimated beat-to-beat interval.
    ∗ To extract the systolic peaks, an initial set of peaks on w0 was used to find a local maximum in each time interval where the filtered waveform w2 was above the baseline *b*.
    ∗ All peaks on w0 were sorted by amplitude in increasing order and selected the amplitude value at the 2/3 length position. Each initial peak needed to satisfy two conditions: it needed to be greater than half of the selected amplitude value, and each peak-to-peak interval could not deviate from the median interval by more than two times, referred to as MAD. MAD was calculated as MAD=median[|t−median(t)|], where *t* is the peak-to-peak intervals. The PPG peaks that did not satisfy these two conditions were identified as potential false peaks, while the remaining were identified as true peaks.
    ∗ An attempt was made to relocate the putative false peaks and identify the locations of true ones. Specifically, starting from each interval’s left-boundary position, median(t) seconds were added, and this point was considered as the expected position of the next peak. Next, the area around the expected position was searched to identify a local maximum on the smoothed waveform w1 within a window size of a length set to MAD. If the local maximum was located at either end of the window, the window size increased by MAD seconds, and the process was repeated. Then, the equivalent maximum was on w0 and labeled as the next peak. Starting from this newly discovered peak position, the procedure above was repeated until the end of the interval was reached and multiple consecutive missed peaks were recovered.
  – **Onset detection:** The onset corresponding to each true peak was detected in three steps, as follows:
    ∗ Ranges were located where w0 was below both the filtered waveform w2 and the baseline *b*.
    ∗ If multiple ranges were identified, they were ranked based on their lengths. Then, the top two ranges’ rightmost one was selected.
    ∗ The minimum position on the waveform w0 in the selected range was considered the onset.
- Method III extracted the systolic peaks based on a block-based method [30], and the local minimum between two successive peaks was defined as the onset.
  – **Systolic peak detection:**
    ∗ A second-order Butterworth 0.5–8 Hz bandpass filter was applied, and then the filter’s output was clipped by keeping the signal above 0 to produce the x[n] signal.
    ∗ The filtered signal was squared to emphasize the large difference between the systolic wave and diastolic wave in x[n]. This squaring step produced the y[n] signal.
    ∗ Blocks of interest were generated based on two event-related moving averages and an offset threshold, as follows:
      (5) $$\left\{\begin{array}{c} {\mathrm{MA}}_{\mathrm{peak}}\left[n\right]=\frac{1}{W_{1}}\left(y\left[n-\left(W_{1}-1\right)/2\right]+\ldots +y\left[n\right]+\ldots +y\left[n+\left(W_{1}-1\right)/2\right]\right)\\ {\mathrm{MA}}_{\mathrm{beat}}\left[n\right]=\frac{1}{W_{2}}\left(y\left[n-\left(W_{2}-1\right)/2\right]+\ldots +y\left[n\right]+\ldots +y\left[n+\left(W_{2}-1\right)/2\right]\right)\\ {\mathrm{THR}}_{1}={\mathrm{MA}}_{\mathrm{beat}}\left[n\right]+\beta \bar{y}\\ {\mathrm{THR}}_{2}=W_{1} \end{array}\right.$$
      where ${\mathrm{MA}}_{\mathrm{peak}}$ is the first moving average used to emphasize the systolic peak area, ${\mathrm{MA}}_{\mathrm{beat}}$ is the second moving average used to emphasize the beat area to be used as a threshold for the first moving average, $\bar{y}$ is the mean of the squaring result, and $W_{1}$, $W_{2}$, and β are parameters. The blocks of interest were generated by comparing the ${\mathrm{MA}}_{\mathrm{peak}}$ signal with ${\mathrm{THR}}_{1}$, and the blocks wider than or equal to ${\mathrm{THR}}_{2}$ were classified as the systolic peak area. The optimized parameters ($W_{1},W_{2},\beta =111\mathrm{ms},\;667\mathrm{ms},\;2$) were used.
    ∗ The maximum value in each block was considered the systolic peak.
  – **Onset detection:** This study did not discuss the onset detection algorithm.

### 2.7. Performance Evaluation

Algorithms were evaluated using two statistical measures:

(6) $$\mathrm{Sensitivity}\;\left(\mathrm{SE}\right)=\frac{\mathrm{TP}}{\mathrm{TP}+\mathrm{FN}}\times 100\%,$$

(7) $$\mathrm{Positive}\;\mathrm{predictivity}\;\left(\mathrm{PP}\right)=\frac{\mathrm{TP}}{\mathrm{TP}+\mathrm{FP}}\times 100\%,$$

where true positive (TP) is the number of features detected as features, false negative (FN) is the number of features that were not detected, and false positive (FP) is the number of non-features that were detected as features. In this study, each feature (systolic peak and onset) obtained by the algorithms was defined as a true positive if it deviated from the corresponding annotation within ±10 ms [31].

## 3. Results

### 3.1. The Graphical User Interface

**Figure 5:**
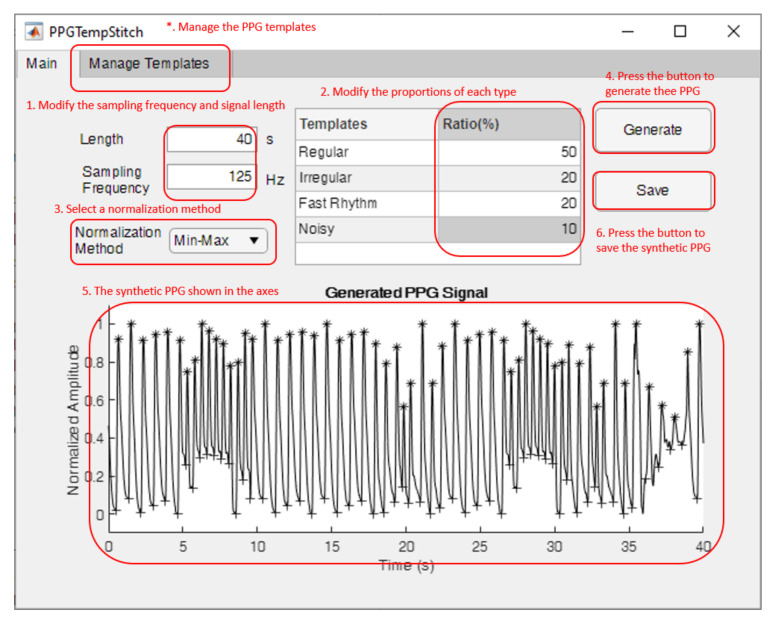
The graphical interface of PPGTempStitch.

Figure 5 shows the main dialogue of the graphical user interface (GUI). Four types of PPG templates were supported in the GUI. These templates were extracted from different subjects in MIMIC III database. To synthesize an annotated PPG, users could complete all their work in the main menu. First, the signal length and sampling frequency of the synthetic PPG were modified. The PPG signal needed be longer than five seconds, the length of all the PPG templates, and so the stitched PPG was resampled to the required sampling rate when the needed sampling frequency was not 125 Hz.

The second step was modifying the percentages of the different PPG types. The sum of all the percentages should be 100. When the percentage of a certain PPG multiplied by the signal length is less than 5 s, this signal type may not appear in the final synthesized PPG. The third step was selecting the normalization method. The toolbox provided two commonly used normalization methods: Min-Max and z-score normalization. When the “Generator” button was pressed, the GUI attempted to generate the synthetic PPG and showed it at the bottom of the dialogue. The toolbox generated PPGs using the following steps:

1. **Determine the proportion of beat types**. The synthesized signal’s length ($n_{s}$) was divided by the template’s length 5 s to obtain the number of templates $n_{at}$. Then, $n_{at}$ was multiplied by the percentage of each template to get the results $n_{atp}$. The integer part of $n_{atp}$ was used as the number of each template. The four templates represented four beat types: regular, irregular, fast rhythm, and noisy.
2. **Randomly arrange the determined number of templates**. Then, the method proposed in this study was used to stitch the determined templates in sequence one by one. When stitching was based on the systolic peak, the stitching point of the first PPG was the last annotated systolic peak, and the stitching point of the second PPG was the first annotated systolic peak. When stitching was based on the onset, the stitching point of the first PPG was the last annotated onset, and the stitching point of the second PPG was the first annotated onset.
3. **Adjust the signal length**. If the length of the synthesized PPG obtained by Step 2 was less than the required length *n*, the difference between the lengths was defined as the new $n_{s}$. Then, Steps 1 and 2 were repeated until the synthesized PPG was longer than *n*, at which point the first *n* seconds of the signal were considered as the signal output $s_{p}$.
4. **Re-sample the signal**. If the desired sampling frequency was not 125 Hz, $s_{p}$ was resampled to the desired frequency to generate the final output $s_{end}$. The annotations were mapped to $s_{end}$ according to the ratio of the required sampling rate to 125. Each annotated systolic peak was corrected to the maximum position in the 0.01 s window centered on the peak. Likewise, the onset was corrected to the minimum position in the 0.01 s window centered on the onset.

After the signals are synthesized, users of the toolbox can press the “Save” button to save the synthetic PPG to a comma-separated values file (.csv), a text file (.txt), a generic data file (.dat), or an Excel spreadsheet file (.xls, .xlsm, or .xlsx). If more noise levels and waveform types are required, users can also add new PPG templates with annotations to the “Manage Templates” tab.

To maintain data consistency, users can only add five-second PPG templates, and the added templates will be resampled at 125 Hz when saving. When the signal length is short, the percentage of each template in the synthesized PPG obtained by this toolbox is not exactly the same as that set by the user.

### 3.2. Performance Evaluation Results

To evaluate a feature extraction algorithm, data containing different conditions are required. Typically, a large number of annotated PPGs are used to evaluate the algorithm’s performance under different conditions. However, this toolbox could generate PPG signals under different conditions, thereby providing a solution to the current shortage of annotated PPGs.

**Figure 6:**
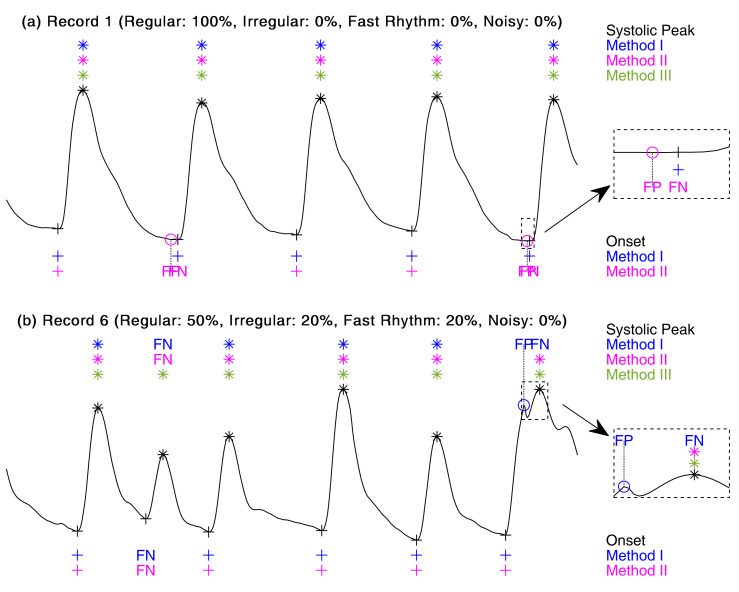
Comparison of the three methods’ results. (**a**) Four-second segment of Record 1. (**b**) Four-second segment of Record 6. The black ‘*’ and ‘+’ refers to the annotated systolic peaks and onsets, respectively. The blue, magenta and dark-green ‘*’ are the true-positive systolic peaks of Methods I, II, and III, respectively. The blue and magenta ‘+’ are the true-positive onsets of Methods I and II, respectively. ‘FP’ and ‘FN’ are the false-positive and false-negative, respectively. The ‘○’ is used to show the location of the false-positive.

To compare the three feature extraction algorithms, 10 PPG records were synthesized using this toolbox. These records contain different conditions. These records contained different conditions: records 1–5 were used to compare these methods’ accuracy in different signal compositions at the same sampling rate and length, records 5–8 were used to compare these accuracies with different sampling rates and lengths under the same signal composition (e.g., the same proportion of beat types), and records 1, 7, and 10 compared these methods under unique conditions. Table 2 compares the application of the three feature extraction algorithms on different synthetic PPGs. Since Method III did not discuss the onset detection algorithm, its onset accuracy was not discussed. Record 1 was stitched only by the regular template. In this case, all three methods got SE=100% and PP=100% in systolic peak detection, and no assessment about which algorithm was best could be made. For onset detection, Method I got SE=100% and PP=100%. However, Method II got SE=61.1% and PP=61.1%. Figure 6a shows a segment of Record 1. Method II failed to detect the second and fifth onsets, and the deviation between the result of Method II and the annotation was greater than 10 ms. When the signal quality decreased, the accuracy of Methods I and II decreased, while Method III still achieved high accuracy.

Comparisons of the three algorithms’ average accuracy revealed that Record 6 had the lowest accuracy. Figure 6b shows a segment of Record 6. Both Methods I and II missed the second systolic peak. Method 1 only selected the region greater than 50% amplitude for Hilbert transform data. This may have been due to the fact that the amplitude of the systolic peak may become lower than the threshold when noisy or irregular events occur. Method II judged the second peak as a false peak that was caused by the peak-to-peak interval threshold.

For the sixth systolic peak, the offset between the detection result of Method I and the annotation was greater than 10 ms. The results of Method III were not exactly the annotation location, but the offset was less than 10 ms. After comparing the average accuracy of 10 records, Method III was found to be the optimal method for detecting the systolic peak. Likewise, the accuracy of Method I was better than Method II for onset detection. Furthermore, by comparing the accuracy of these 10 records, the changes in sampling frequency were found to have no great influence on the three methods’ accuracy.

## 4. Discussion

After comparing the average of the three algorithms’ accuracy on different records, the following ratio is recommended to test the feature extraction algorithm: the proportions of regular, irregular, fast rhythm, and noisy beats should be 50%, 20%, 20%, and 10%, respectively. Users of the toolbox can synthesize more PPGs with different sampling rates and different waveforms to evaluate the feature extraction algorithms. Because the order of the templates is random, the toolbox’s PPG signal output may differ even if the same parameters (e.g., scale, sampling rate, signal length) are used.

To ensure the authenticity of the synthetic PPGs, all PPG templates came from real PPGs in the MIMIC III database. Noise was not added to the PPG templates. If noise is required, users can generate PPGs and then add noise. The advantage of this toolbox is that it can generate PPGs with annotations; previous studies [15,16,17,18,19] only generated the PPGs without annotation. Thus, by using this toolbox, users can generate PPGs to test and compare the developed feature extraction algorithms. Several PPG features exist in the time domain—onset, systolic peak, dicrotic notch, and diastolic peak. This study focused only on the main features of a PPG waveform: systolic peak and onset. However, other annotated features can also be generated using the same method, as discussed in [32,33].

One advantage of this toolbox is that the augmented PPGs are generated based on human data. Augmenting PPGs under the same condition can mislead the evaluation of the feature extraction algorithms. However, the combination of different data types covers different conditions to simulate real-life situations, reducing bias in performance assessment. One limitation of this toolbox is that only four types of PPG templates are included by default. If more PPG morphologies are needed, users can add their templates in the “ManageTemplates” tab of the toolbox.

## 5. Conclusions

PPGTempStitch, a new MATLAB toolbox for generating annotated PPG signals with any sampling frequency and a time length longer than 5 s, was described herein. The generated PPGs can contain different noise, waveform, and heart rhythm levels to simulate real-life conditions. Users can generate different annotated PPG types by adding new PPG templates. The developed toolbox is free and open-source software. Hopefully, the user-friendly toolbox will make PPG research easier, especially when evaluating PPG feature extraction algorithms.

## Figures and Tables

**Table 1:** Signal quality indices of the stitching results (“stitching beat” refers to a beat that has been stitched, “previous beat” to the beat prior to the stitching beat, and “next beat” to the beat after the stitching beat. The “signal quality index” refers to skewness of the beat).

First PPG	Second PPG	Based on Systolic Peak				Based on Onset			
		Previous Beat	Stitching Beat	Next Beat	S1	Previous Beat	Stitching Beat	Next Beat	S2
Regular	Regular	0.64	0.66	0.67	1.97	0.64	0.66	0.67	1.97
	Irregular	0.64	0.97	0.96	2.57	0.62	0.97	0.54	2.12
	Fast Rhythm	0.64	0.46	0.69	1.80	0.59	0.47	0.69	1.76
	Noisy	0.64	−0.19	0.00	0.45	0.59	0.10	1.03	1.72
Irregular	Regular	1.08	0.66	0.67	2.41	1.08	0.66	0.67	2.41
	Irregular	1.08	0.92	0.96	2.97	1.07	0.98	0.54	2.59
	Fast Rhythm	1.08	0.49	0.69	2.27	1.02	0.53	0.69	2.25
	Noisy	1.08	−0.61	0.00	0.46	1.02	0.10	1.03	2.15
Fast Rhythm	Regular	0.58	0.72	0.67	1.96	0.71	0.64	0.67	2.01
	Irregular	0.58	0.99	0.96	2.53	0.72	0.96	0.54	2.22
	Fast Rhythm	0.58	0.78	0.69	2.05	0.65	0.81	0.69	2.15
	Noisy	0.58	0.35	0.00	0.93	0.62	0.07	1.03	1.72
Noisy	Regular	0.88	0.36	0.67	1.91	−1.37	0.62	0.67	-0.08
	Irregular	0.88	0.24	0.96	2.09	−0.98	0.95	0.54	0.50
	Fast Rhythm	0.88	0.63	0.69	2.21	0.89	0.71	0.69	2.30
	Noisy	0.88	−0.29	0.00	0.59	0.79	0.04	1.03	1.86

**Table 2:** Comparison of the accuracy of the feature extraction algorithms on different synthetic PPGs. ‘SE’ refers to the sensitivity of the algorithm, while ‘PP’ refers to the positive predictivity.

							Method I [28]				Method II [29]				Method III [30]		
	**Sampling**	**Length**	**PPG Templates Ratio (%)**				**PPG Onsets**		**PPG Systolic Peaks**		**PPG Onsets**		**PPG Systolic Peaks**		**PPG Systolic Peaks**		
	**Frequency**	**(s)**															
			**Regular**	**Irregular**	**Fast**	**Noise**	**SE**	**PP**	**SE**	**PP**	**SE**	**PP**	**SE**	**PP**	**SE**	**PP**	**Average**
					**Rhythm**		**(%)**	**(%)**	**(%)**	**(%)**	**(%)**	**(%)**	**(%)**	**(%)**	**(%)**	**(%)**	
Record 1	125	60	100	0	0	0	100	100	100	100	61.1	61.1	100	100	100	100	92.22
Record 2	125	60	90	10	0	0	98.6	100	98.6	100	63.5	64.4	98.6	100	100	100	92.37
Record 3	125	60	80	10	10	0	93.8	98.7	96.2	100	61.3	68.1	91.1	100	100	100	90.92
Record 4	125	60	70	10	10	10	88.8	97.3	91.3	98.6	65	71.2	91.3	100	97.5	98.7	89.97
Record 5	125	60	50	20	20	10	85.4	95	89.9	98.8	59.6	74.6	80	100	100	100	88.33
Record 6	125	300	50	20	20	10	83.4	96.1	86.6	98.2	60	74.2	80.7	99.7	98.4	98.6	87.59
Record 7	200	300	50	20	20	10	85.9	97.4	87.1	98.5	67.7	83.7	80.3	99.4	98.9	99.1	89.8
Record 8	400	600	50	20	20	10	78.4	98	87.9	100	68.4	84.6	79.1	97.8	99	99.1	89.23
Record 9	125	600	70	10	10	10	87.5	96.2	90.9	98.1	62.2	69.5	89.5	100	98.8	98.8	89.15
Record 10	400	600	70	10	10	10	84.8	98	90.8	100	72.8	81.3	89.5	100	98.5	98.5	91.42
Average	-	-	-	-	-	-	88.66	97.67	91.93	99.22	64.16	73.27	88.01	99.69	99.11	99.28	

## Data Availability

The PPGTempStitch MATLAB toolbox is publicly available and can be downloaded via this link: https://github.com/Elgendi/PPG-Template-Stitching, accessed on the 8 June 2021.
